# The Role of Angiopoietin-2 in Post-Burn Pneumonia

**DOI:** 10.3390/ebj7010001

**Published:** 2025-12-19

**Authors:** Mary Grace Murray, Ryan M. Johnson, Abigail B. Plum, Natalia Carbajal Garcia, Kevin E. Galicia, Alexandra Brady, Madison Kipp, Irene B. Helenowski, Madison M. Tschann, Connor Guzior, Richard P. Gonzalez, Mashkoor A. Choudhry, John C. Kubasiak

**Affiliations:** 1 Department of Surgery, Loyola University Medical Center, Maywood, IL 60153, USA; mmurray14@luc.edu (M.G.M.); ncarbajal@luc.edu (N.C.G.); kgalicia@luc.edu (K.E.G.); mchoudhry@luc.edu (M.A.C.); 2 Burn and Shock Trauma Research Institute, Loyola University Chicago, Maywood, IL 60153, USA; aplum@luc.edu (A.B.P.);; 3 Clinical Research Office, Loyola University Chicago, Maywood, IL 60153, USA; 4 Department of Microbiology and Immunology, Loyola University Chicago, Maywood, IL 60153, USA

**Keywords:** burn, angiopoietin, pneumonia, lung, inflammation, neutrophil

## Abstract

Background: Pneumonia contributes to post-burn morbidity and mortality. Understanding the mechanisms that predispose burn patients to pneumonia is crucial to both stratifying patients at increased risk and developing targeted interventions. Methods: A prospective observational study was conducted with 47 human patients who sustained large burn injuries with serum collected on days 2 and 3 post-burn and assessed for Angiopoietin-1 (Ang-1) and -2 (Ang-2). C57BL/6 mice were subjected to either sham injury or a 12.5% total body surface area (TBSA) scald burn injury, and plasma and lungs were assessed. Results: Patients who developed pneumonia within 30 days of injury had higher serum Ang-2 and Ang-2/1 ratio on post-injury days 2 and 3. Similar to patient findings, we observed an increase in Ang-2 in burn mice compared to sham. Within the lungs of burn mice, we found significant increases in Tyrosine kinase with immunoglobulin and epidermal growth factor homology domains 2 (TIE2) receptor transcript Tek, downstream mediators TNFAIP3 Interacting Protein 2 (Tnip2) and phosphoinositide-3-kinase regulatory subunit 1 (Pik3r1), in addition to endothelial adhesion molecules intracellular adhesion molecule-1 (ICAM-1) and vascular cell adhesion molecule-1 (VCAM-1), along with neutrophil infiltration and markers compared to sham. Conclusions: These findings suggest that burn injury increases Angiopoetin-2 and downstream signaling in the lungs, which may contribute to post-burn pulmonary dysfunction. Further studies are necessary to understand if modulating the Ang–TIE2 axis can protect against pneumonia post-burn.

## 1. Introduction

Burn injury results in high morbidity and mortality, with approximately 180,000 deaths globally each year [1]. There is a higher incidence of burn injury in men than women, with a ratio of 2.06:1 adult men to women, respectively [2]. Pneumonia is a significant complication after burn, observed in 48% of patients after large burn, and contributes to 25% of burn mortality [3]. The American Burn Association 2016 National Burn Repository report found that pneumonia was the most frequent clinical complication in burn patients in the United States [4]. Patients with large burns often require mechanical ventilation, which places them at risk for ventilator-associated pneumonia (VAP) [4], remaining a significant concern for critically ill burn victims. Current pneumonia therapies in the Burn ICU are aimed at early recognition and treatment with antibiotics [5]. A VAP Prevention Bundle has shown efficacy in reducing the incidence of VAP in critically ill burn patients [6] but, currently, targeted treatments or prevention strategies targeting the underlying pathophysiology are not in use. There are currently no pharmacologic interventions that would allow for the prophylactic treatment of at-risk patients for the development of pneumonia in critical illness after burn injury. The rise of multidrug-resistant organisms and prevalence of mortality from pneumonia in critically ill burn patients [7] necessitate better biomarkers for prognostication and preclinical investigation into future drug targets to reduce the incidence of pneumonia in this population.

Recent studies from the Hofmaenner group in Switzerland as well as our laboratory in the United States have demonstrated an increased risk of mortality in burn patients predicted by both elevations in serum Ang-2 as well as an elevated Ang-2/1 ratio [8,9]. Additionally, the CAPNETZ and PROGRESS study groups have identified increases in Angiopoietin-2 (Ang-2) and decreases in Angiopoietin-1 (Ang-1) to be predictive of 28-day mortality in patients with community acquired pneumonia [10]. The pulmonary vascular endothelium plays a key role in regulating pulmonary inflammation, fluid leak, and bacterial susceptibility [11,12]. Ang-1 and Ang-2 are key regulators of endothelial inflammation, permeability, and cell-cycle progression [13]. Briefly, Ang-1 agonizes the Tyrosine kinase with immunoglobulin and epidermal growth factor homology domains 2 (TIE2) receptor and stabilizes vascular endothelium by increasing tight junction expression [14,15] and reducing leukocyte adhesion molecules including intracellular adhesion molecule-1 (ICAM-1) and vascular cell adhesion molecule-1 (VCAM-1) [16]. In contrast, Ang-2 competes for TIE2 binding, where it acts primarily in an antagonistic manner, resulting in vascular destabilization by decreasing tight junctions between endothelial cells [17].

ICAM-1 and VCAM-1 are both expressed on vascular endothelium and play roles in the recruitment of leukocytes, particularly neutrophils, to tissue [18,19]. ICAM-1 is expressed most abundantly within pulmonary endothelium compared to other organs [20]. In the context of neutrophil tethering, rolling, adhesion, and ultimate transendothelial migration, ICAM-1 and VCAM-1 both bind to ligands on neutrophils and facilitate firm adhesion to the endothelial wall [21]. We and others have characterized marked neutrophil chemotaxis, infiltration, pulmonary congestion, and robust pulmonary inflammation in lung tissue within 6–24 h after burn in a murine model [22,23,24]. While neutrophils play a vital role in host immunity against invading pathogens, overactivation of neutrophils can lead to detrimental tissue damage and can drive progression of severe pneumonia, which is often seen in burn patients who go on to develop pneumonia during their hospital stay [3,25]. In addition to neutrophil recruitment by upregulation of endothelial expression of ICAM-1 and VCAM-1, neutrophils express the TIE2 receptor and can respond to angiopoietin stimulation to further increase tethering to endothelium [26]. Additionally, both Ang-1 and Ang-2 have been shown to each induce the formation of Neutrophil Extracellular Traps (NETosis) via TIE2 signaling [27], which can contribute both to pathogen neutralization but also exacerbate tissue damage and further inflammation [28]. The combination of upregulation of ICAM-1 and VCAM-1 along with destabilization of endothelial tight junctions and barrier integrity induced by Ang-2 via the TIE2 receptor indicates that this pathway may contribute to neutrophil infiltration following burn.

This study aims to determine if early Ang-2/1 imbalance is predictive of 30-day development of pneumonia and to utilize a murine model of burn injury to evaluate downstream pathways within the lung.

## 2. Materials and Methods

### 2.1. Study Population

This study was approved by the Loyola University Chicago Institutional Review Board (IRB #215735). All patients 18 and older who presented to the Loyola Medicine Burn Center after thermal burn injury were screened for enrollment. Informed consent was obtained from all participants or their legal authorized representatives. All adult patients who sustained burn injuries greater than or equal to 5% TBSA were prospectively enrolled. Patients were pragmatically enrolled, as two periods of enrollment freeze were initiated during this study and coordinator staffing was unavailable. Exclusion criteria included prior cancer, hematologic or immunologic diseases, concurrent injury, or if mortality was expected within 24 h of admission based on the clinical judgment of the treating attending physician. Between November 2022 and November 2024, a total of 62 patients met the inclusion criteria and provided consent for enrollment. Due to clinical factors, for example, urgent OR, 35 patients had serum collected from post-burn day 2 (PBD2) and 35 patients had serum from post-burn day 3, with 23 patients having serum collected from both PBD2 and PBD3; this resulted in 47 total patients in the analysis.

### 2.2. Clinical Patient Data

Patient demographics, laboratory results, and treatment data were prospectively collected from medical records for all patients included in the study. Demographic data included age, gender, race, ethnicity, and body mass index (BMI). Clinical data included %TBSA, Baux score without inhalation injury (II), length of hospital and ICU stay, need for mechanical ventilation, and the number of ventilator days for patients requiring mechanical ventilation, based on definitions from the Burn Care Quality Platform (BCQP) Registry data dictionary. Patients with secured airway and concern for inhalation injury, as judged by the attending burn surgeon, underwent diagnostic bronchoscopy and were graded for inhalation injury as negative (0) or positive grades 1–4. The classification system in use at the Loyola University Medical Center Burn Unit follows the abbreviated injury score (AIS) for inhalation injury [29]. Therapeutic heparin and mucomyst nebulizers are provided to grades 3–4. Based on the AIS criteria, inhalation injury (II) was diagnosed and graded based on bronchoscopy findings, with criteria as follows: grade 0: negative for soot, erythema, edema, bronchorrhea, or obstruction; grade 1: minor/patchy soot, erythema, bronchorrhea, or obstruction; grade 2: moderate soot, erythema, bronchorrhea, or obstruction; grade 3: severe inflammation with friability, copious soot deposits, bronchorrhea or obstruction; and grade 4: mucosal sloughing, gross necrosis, or endoluminal obstruction [29]. There was no statistical difference between pneumonia development in this cohort based on inhalation injury; patients with inhalation injury who did not develop pneumonia had an average II severity grade of 1.6 (range 1–3), while those with II who did develop pneumonia within 30 days had an average severity grade of 1.25 (range 1–2).

### 2.3. Sample Collection

Peripheral blood samples were collected on post-burn days 2 and 3 (PBD) in Serum Separation Tubes (SST) (BD Biosciences, Franklin Lakes, NJ, USA) after the patient had undergone approximately 24 h of resuscitation (2 mL × body weight in kg × %TBSA burned) followed by centrifugation at 2500× *g* for 10 min according to manufacturer recommendations to isolate serum. Aliquots were generated and frozen at −80 °C until batch analysis.

### 2.4. Serum Biomarker Measurements

Serum Ang-1 and Ang-2 levels were quantified using pre-coated ELISA kits (Ang-1: Cat. EHANGPT1; Ang-2: Cat. KHC1641; ThermoFisher, Waltham, MA, USA), following the manufacturer’s protocols at a 1:10 dilution. Absorbance at 450 nm was measured using SpectraMax iD5 plate reader (Molecular Devices, San Jose, CA, USA).

### 2.5. Animals

Male C57BL/6 mice (8–10 weeks old) were obtained from Charles River Laboratories and maintained in AAALAC-accredited animal housing facilities at Loyola University Chicago Health Sciences Division, Maywood, IL, USA. The murine samples in this study were generated in June 2023. The identification number for the animal care and use protocol used in this study is IACUC 2021014.

### 2.6. Murine Model of Burn Injury

We utilized a well-established model of scald burn injury as previously described [30,31]. Briefly, mice were pre-treated with ~1 mg/kg of buprenorphine subcutaneously one hour prior to injury. Mice were anesthetized with ketamine hydrochloride (~80 mg/kg) and xylazine (~1.2 mg/kg) by intraperitoneal injection. The dorsal surfaces of mice were shaved and they were placed into a prefabricated template exposing ~12.5% TBSA calculated using Meeh’s formula [32]. Mice receiving burn injury were lowered into an ~85 °C water bath for ~7 s to induce a full-thickness scald burn injury. Sham animals were lowered into a 37 °C water bath for ~7 s. Immediately following burn or sham injury, animals were gently dried and administered a 1.0 mL normal saline resuscitation by intraperitoneal injection. Animals were returned to their cages with food and water ad libitum and monitored during anesthesia recovery. All animal experiments were conducted in accordance with the guidelines set forth in the Animal Welfare Act and were approved by the Institutional Animal Care and Use Committee at Loyola University Health Sciences Division.

### 2.7. Mouse Enzyme-Linked Immunosorbent Assay (ELISA)

One day after sham or burn injury, mice were deeply anesthetized with isoflurane, and heparinized blood was drawn via cardiac puncture. Mice were euthanized and lungs were harvested and snap frozen for downstream analysis. Heparinized blood was spun at 2000× *g* in a precooled 4 °C centrifuge for 15 min. Plasma was collected and stored at −80 °C until analysis. Plasma Angiopoietin-2 levels were determined using Mouse & Rat Angiopoietin-2 Quantikine ELISA Kit (R&D Systems Cat. MANG20) using a 1:40 dilution of plasma in technical duplicate and reported as ng of Ang-2/mL of plasma. Absorbance at 450 nm was measured using SpectraMax iD5 plate reader (Molecular Devices).

### 2.8. RNA Isolation and RT-qPCR

To evaluate mRNA expression of Ang–TIE2 signaling pathway markers and neutrophil mediators, reverse transcription–quantitative polymerase chain reaction (RT-qPCR) was performed on lung tissue from murine samples. Briefly, RNA was isolated from snap-frozen lung tissue using mirVana miRNA Isolation Kit (ThermoFisher Cat. AM1561) and phenol according to the manufacturer’s instructions. RNA concentration and purity were assessed via NanoDrop 2000 spectrophotometer (Thermo Scientific). cDNA synthesis was performed using High-Capacity cDNA Reverse Transcription Kit (ThermoFisher Cat. 4374967) according to the manufacturer’s instructions. qPCR was performed using TaqMan Fast Advanced Master Mix (ThermoFisher Cat. 4444965) with predesigned TaqMan primer probes for Ang–TIE2 pathway components along with neutrophil mediators and markers (Appendix A Table A1) using GAPDH as housekeeping control (ThermoFisher Cat. 4352339E). qPCR data were analyzed using 2^−ΔΔCt^ with the average ΔCt of sham mice.

### 2.9. Histology

At the time of collection, lungs were inflated with 10% phosphate-buffered formalin by injection into the bronchus. Samples were fixed in formalin overnight and then transferred to 70% ethanol to AML laboratories (Saint Augustine, FL, USA) for embedding and sectioning. AML laboratories performed hematoxylin and eosin (H&E) staining. Immunohistochemistry was performed for Angiopoetin-2. Slides were deparaffinized and rehydrated as previously described [33]. Samples were permeabilized for 30 min in 95 °C 10 mM Sodium Citrate pH 6.0 and blocked with BlockAid (ThermoFisher Cat. B10710). Slides were stained with an Anti-Angiopoietin-2 antibody (ThermoFisher Cat. PA5-23612) at a 1:1000 dilution in BlockAid overnight at 4 °C and washed in Tris Buffered Saline with Tween 20 (TBST). Secondary HRP-linked SignalStain^®^ Boost IHC Detection Reagent was incubated for 1 h at room temperature. Slides were developed with DAB (ThermoFisher Cat. 34065).

### 2.10. Statistics

Graphs were generated and statistics were calculated using GraphPad Prism 10 and R 4.3.1. Patient demographic data for patients who did not and did develop pneumonia within 30 days of burn were assessed using Wilcoxon rank sum test, Fisher’s exact test, and Wilcoxon rank sum exact test where appropriate, as indicated in Table 1. For patient Angiopoietin data, values were assessed via ROUT with a Q = 1% and statistical outliers were removed. Outliers removed from Ang-2 were subsequently removed from the Ang-2/1 ratio. Differences between patients who did or did not develop pneumonia within 30 days of burn were assessed using Mann–Whitney U-test. Multivariable logistic regression adjusted for age, TBSA, and II was performed on patient samples. Differences between murine samples were analyzed with unpaired *t*-tests or simple linear regression. Data, wherever applicable, are presented as means ± SEM; linear regressions are plotted with 95% confidence intervals. Unless otherwise noted, significance is represented as follows: * *p* < 0.05, ** *p* < 0.01, *** *p* < 0.001, and **** *p* < 0.0001.

## 3. Results

### 3.1. Patient Demographics and Injury Characteristics

A total of 62 patients met the criteria and provided consent for enrollment. Among those, 35 patients had serum from post-burn day 2 (PBD2) and 35 patients had serum from post-burn day 3, with 23 patients having serum collected from both PBD2 and PBD3, resulting in 47 total patients in the analysis. As reflected in Table 1, 10 patients (21.28%) developed pneumonia within 30 days of injury. There was no difference in age, sex, race, ethnicity, and BMI between those who did not and did develop pneumonia. Total body surface area (TBSA) was greater in those who developed pneumonia than those that did not (*p* < 0.05), and there were a greater proportion of patients with TBSA >20% who developed pneumonia (*p* < 0.05). Baux score without inhalation injury was higher in pneumonia (*p* = 0.051). The number of patients with inhalation injury (II) in this cohort was only nine patients, four of whom developed pneumonia within 30 days, thus there was not a significant difference in terms of pneumonia development based on II in this study (*p* = 0.081), likely due to the small number of II patients included. Patients who developed pneumonia had longer length of stays (LOS) overall and in the intensive care unit (ICU) (*p* < 0.05). Not surprisingly, patients who went on to develop pneumonia within the first 30 days had a significantly higher need for mechanical ventilation (*p* < 0.001); however, for those patients that required mechanical ventilation, there was no difference between days of ventilation regardless of pneumonia development (*p* = 0.238) (Table 1).

**Table 1:** Demographics and injury characteristics.

Characteristics	Pneumonia Development ≤ 30 Days			*p* Value *
	All	Negative	Positive	
No. of Patients, No. (%)	47 (100%)	37 (78.72%)	10 (21.28%)	
Age, years, median (IQR)	44 (35–57)	42 (32–57)	47 (38.25–52.75)	0.594
Sex, No. (%)				>0.999
Male	36 (76.60%)	28 (75.68%)	8 (80.00%)	
Female	11 (23.40%)	9 (24.32%)	2 (20.00%)	
Race, No. (%)				0.292
Asian	2 (5.26%)	2 (5.41%)	-	
Black	7 (14.89%)	4 (10.81%)	3 (30.00%)	
Caucasian	24 (51.06%)	18 (48.65%)	6 (60.00%)	
Other	13 (27.66%)	12 (32.43%)	1 (10.00%)	
Unknown	1 (2.13%)	1 (2.70%)	-	
Ethnicity, No. (%)				0.238
Hispanic or Latino	13 (27.66%)	12 (25.53%)	1 (10.00%)	
BMI, median (IQR)	28.10 (23.87–33.00)	28.30 (24.90–32.91)	26.57 (22.10–33.15)	0.567
TBSA%, median (IQR) (Min–Max)	15.75 (12.00–35.00) (5.00–72.00)	15.00 (10.50–26.00) (5.00–72.00)	34.50 (19.25–58.75) (12.00–70.00)	**0.013**
TBSA >20%, No. (%)	20 (42.55%)	12 (32.43%)	8 (80.00%)	**0.011**
Baux Score (Without II), median (IQR)	71.2 (53.00, 86.00)	66.00 (50.00, 85.00)	79.50 (68.50, 100.5)	0.051
Inhalation Injury (II), No. (%)	9 (19.15%)	5 (13.51%)	4 (40.00%)	0.081
LOS (days), median (IQR)	19.00 (11.00–37.00)	15.00 (11.00–25.00)	43.00 (25.00–63.25)	**0.020**
ICU LOS (days), median (IQR)	15.00 (10.00–36.00)	12.00 (7.00–25.00)	43.00 (19.75–63.50)	**0.034**
Mechanical Ventilator, No. (%)	20 (42.55%)	11 (29.73%)	9 (90.00%)	**<0.001**
Ventilator Days, median (IQR)	18.50 (4.00–48.25)	10.00 (3.00–29.00)	22.00 (15.00–49.00)	0.238
Serum Ang-1 (ng/mL), n, median, (IQR)				
PBD2	n = 35, 4.986 (2.315–6.501)	n = 25, 5.248 (2.575–7.433)	n = 10, 4.455 (1.712–5.746)	0.255
PBD3	n = 33, 5.121 (3.838–7.177)	n = 26, 5.332 (4.281–7.971)	n = 7, 3.838 (2.248–5.995)	0.268
Serum Ang-2 (ng/mL), n, median (IQR)				
PBD2	n = 31, 2.214 (1.451–2.907)	n = 21, 1.558 (1.369–2.810)	n = 10, 3.152 (2.310–4.353)	**0.015**
PBD3	n = 30, 1.881, (1.451–2.907)	n =23, 1.805 (1.176–2.082)	n = 7, 4.145 (3.546–6.794)	**<0.001**
Serum Ang-2/1 ratio, n, median (IQR)				
PBD2	n = 31, 0.495 (0.254–1.176)	n = 21, 0.298 (0.234–0.660)	n =10, 0.991 (0.574–1.953)	**0.007**
PBD3	n = 30, 0.400 (0.244–0.695)	n = 23, 0.317 (0.221–0.497)	n = 7, 1.700 (0.510–2.786)	**0.002**

* Wilcoxon rank sum test; Fisher’s exact test; Wilcoxon rank sum exact test. Significant *p* values (*p* < 0.05) are bolded. Angiopoietin-1 (Ang-1), angiopoietin-2 (Ang-2), body mass index (BMI), intensive care unit (ICU), inhalation injury (II), interquartile range (IQR), length of stay (LOS), post-burn day (PBD), total body surface area (TBSA). Bold format represents the significant data.

### 3.2. Early Elevation of Ang-2 and the Ang-2/1 Ratio Are Significantly Higher in Patients Who Develop Pneumonia Within 30 Days of Burn

Early (post-burn day 2 and 3) increases in Ang-2 and an elevation in the Ang-2/1 ratio are predictive of 30-day mortality in burn patients [8,9]. As pneumonia is the most frequent clinical complication after burn [4] and ultimately contributes to 25% of burn-related mortality [3], we hypothesized that Ang-2 may contribute to pneumonia incidence in this population.

**Figure 1:**
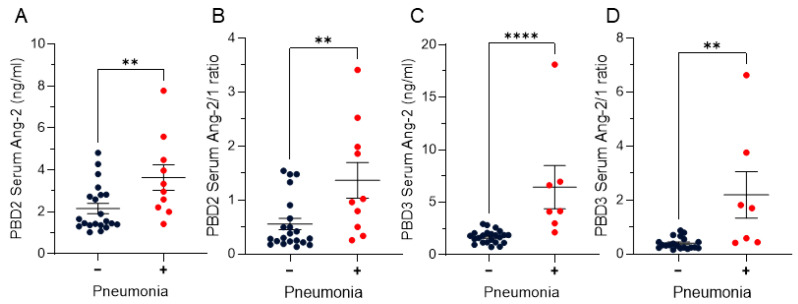
Early elevation of Angiopoietin-2 and Ang-2/1 ratio in patient serum correlates with 30-day mortality. Burn patients were prospectively enrolled and serum was isolated and evaluated via ELISA for Angiopoietin-1 and 2. Dark blue dots represent patients who did not develop pneumonia within 30 days of burn, and red dots represent patients who did develop pneumonia within 30 days of burn. (**A**) Post-burn day 2 (PBD2) serum Ang-2 and (**B**) PBD2 Ang-2/1 ratio for patients that did not (−) and did (+) develop pneumonia within 30 days of injury ((**A**) *p* < 0.05, (**B**) *p* < 0.01). (**C**) Post-burn day 3 (PBD3) serum Ang-2 and (**D**) PBD3 Ang-2/1 ratio ((**C**) *p* < 0.0001, (**D**) *p* < 0.01). Significance was analyzed via Mann–Whitney U-test, ** *p* < 0.01, **** *p* < 0.0001, PBD2 (−) n = 21, (+) n = 10, PBD3 (−) n = 23, (+) n = 7.

There were no significant differences on PBD2 or PBD3 in Ang-1 in patients who did or did not develop pneumonia within 30 days of injury (Table 1). Elevated serum Ang-2 levels on post-injury day 2 were significantly higher in patients who developed pneumonia (*p* < 0.01) (Figure 1A). On day 3, Ang-2 levels remained significantly higher in this cohort (*p* < 0.0001) (Figure 1B). Additionally, a higher Ang-2/1 ratio on both days 2 (*p* < 0.01) and 3 (*p* < 0.01) were associated with the development of pneumonia (Figure 1C,D).

### 3.3. Increased Ang-2 in Murine Model of Burn Injury

In multivariable logistic regression adjusted for age, TBSA, and inhalation injury, higher Ang-2 on PBD2 and PBD3 was not significantly associated with increased odds of pneumonia. Using log_2_-transformed predictors, PBD2 Ang-2 showed an odds ratio (OR) of 5.229 (95% CI 1.109–39.096; *p* = 0.0585) and PBD3 Ang-2 had an OR of 100.639 (95% CI 3.278–102,009.561; *p* = 0.0590). Ang-1 at both time points was not associated with pneumonia, with a 95% CI spanning one at both time points. Ang-2/Ang-1 ratios were not significantly associated with pneumonia odds at both PBD2 OR = 2.255 (95% CI 0.883–6.805, *p* = 0.106) and PBD3 OR = 4.696 (95% CI 1.389–48.967, *p* = 0.0792) (Table 2).

**Figure 2:**
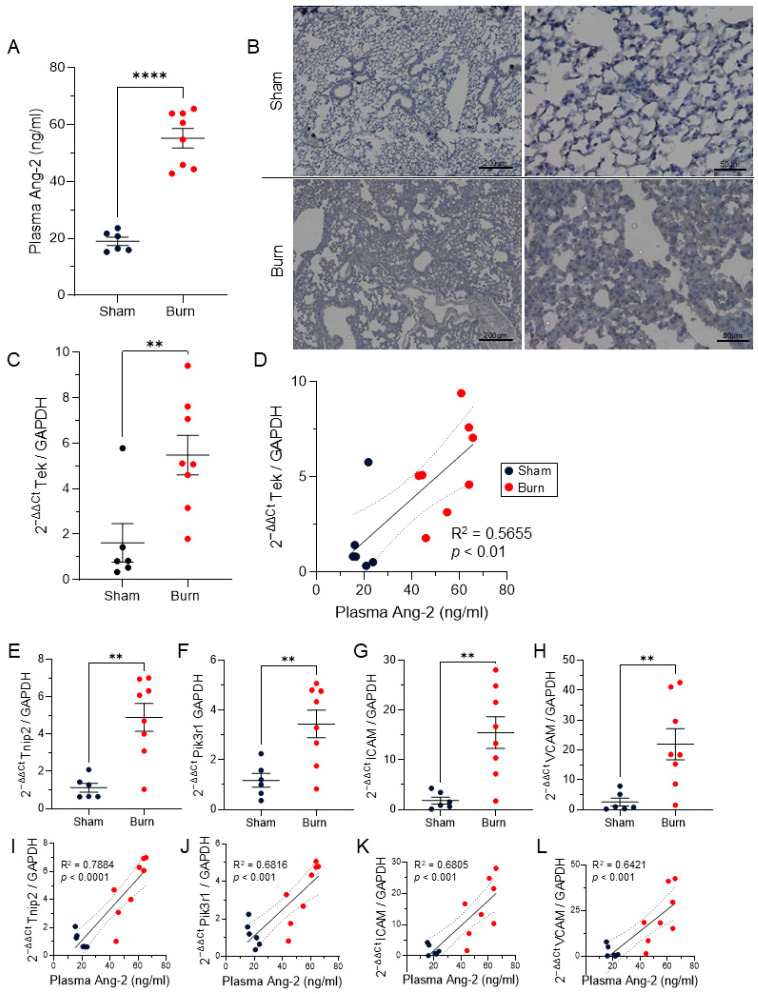
Elevated plasma Angiopoietin-2 and downstream pulmonary mediators in a murine model of burn injury. Heparinized plasma was collected one day after burn or sham injury and evaluated for Ang-2 via ELISA. Dark blue dots represent mice that received sham injury, red dots represent mice that received burn injury. (**A**) Plasma Ang-2 was significantly increased in burn mice compared to sham. (**B**) Representative IHC of Ang-2 in sham and burn lungs. (**C**–**H**) Lung tissue was flash frozen one day after burn injury and then isolated for RNA, and RT-qPCR was performed with GAPDH as housekeeping control. (**C**) RT-qPCR expression of Tek, the transcript for the TIE2 receptor, was significantly elevated in burn lungs compared to sham (*p* < 0.01). (**D**) Tek expression positively correlated with plasma Ang-2 levels (simple-linear regression, R^2^ = 0.5655, *p* < 0.01). (**E**) Tnip2, the transcript of ABIN2, and (**F**) Pik3r1, a subunit of PI3K, were both significantly upregulated in the lungs following burn injury. (**G**) ICAM-1 and (**H**) VCAM-1 were also both significantly upregulated in the lungs following burn injury. For two-group analysis, significance was analyzed by unpaired *t*-test, n = 6–8 animals per group, ** *p* < 0.01, **** *p* < 0.0001. (**I**–**L**) Lung expression of Tnip2, Pik3r1, ICAM-1, and VCAM-1 all positively correlated with plasma Ang-2 levels (simple-linear regression, *p* < 0.001).

Due to elevations in Ang-2 being associated with pneumonia in our patient cohort, we hypothesized that Ang-2 would also be elevated in mice that received burn injury compared to sham injury. Our results indicate that, in mice that received a 12.5% TBSA burn injury, plasma Ang-2 was significantly elevated compared to those that received a sham injury (*p* < 0.0001, n = 6–8) (Figure 2A). Furthermore, immunostaining demonstrates diffuse Ang-2 localization throughout the lungs of mice subjected to burn (Figure 2B).

### 3.4. Pulmonary TIE2 Signaling Post-Burn

Ang-2 primarily signals through the TIE2 receptor expressed on the endothelium [13]. We evaluated the expression of Tek, the transcript for TIE2, and found an increase in Tek expression in the lungs of mice that sustained burn compared to sham injury (*p* < 0.01) (Figure 2C). Additionally, we found that plasma levels of Ang-2 significantly positively correlated with pulmonary levels of Tek expression (R^2^ = 0.5655, *p* <0.01) (Figure 2D).

Ang-2 acts on TIE2, which signals through both phosphoinositide 3-kinase (PI3K) and A20-binding inhibitor of NF-kb 2 (ABIN2) [34]. ABIN2 is encoded by TNFAIP3 Interacting Protein 2 (Tnip2), which was significantly increased in the lungs of mice receiving burn injury compared to sham (*p* < 0.01) (Figure 2E). Similarly, expression of phosphoinositide-3-kinase regulatory subunit 1 (Pik3r1), which encodes a subunit of phosphoinositide-3-kinase (PI3K), was also significantly increased in the lungs following burn injury (*p* < 0.01) (Figure 2F). Pulmonary expression of both Tnip2 and Pik3r1 strongly positively correlated with plasma Ang-2 (Tnip2 R^2^ = 0.7884, *p* < 0.0001; Pik3r1 R^2^ =0.6816, *p* < 0.001) (Figure 2I,J).

In healthy vessels, Ang-1 stimulation of TIE2 leads to repression of Ang-2, ICAM-1, and VCAM-1 transcription [16,34]. When high levels of Ang-2 are present, Ang-2-mediated TIE2 signaling leads to a positive feedback loop of increased Ang-2 transcription, as well as ICAM-1 and VCAM-1 expression [34]. Indeed, in the lungs of mice subjected to burn injury, there were increases in ICAM-1 and VCAM-1 (*p* < 0.01, Figure 2G,H). The levels of ICAM-1 and VCAM-1 also both strongly positively correlated with plasma Ang-2 (ICAM-1 R^2^ = 0.6805; *p* < 0.001, VCAM-1 R^2^ = 0.6421, *p* < 0.001) (Figure 2K,L).

### 3.5. Pulmonary Neutrophil Infiltration

**Figure 3:**
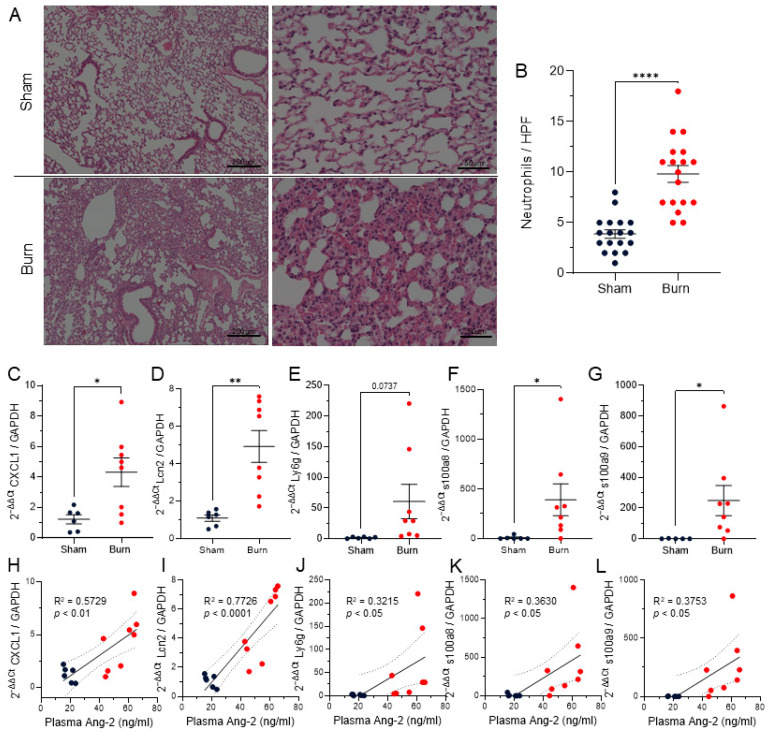
Pulmonary neutrophil infiltration correlates with plasma Angiopoietin-2. Dark blue dots represent mice that received sham injury, red dots represent mice that received burn injury. (**A**) Representative H&E of lungs of sham and burn mice. (**B**) Neutrophils were counted in 6 high-powered fields in 3 mice each of sham and burn (*p* < 0.0001). (**C**) Lung RT-qPCR of chemokine Cxcl1 was elevated in burn compared to sham (*p* < 0.05) and (**H**) positively correlated with plasma Ang-2 (*p* < 0.01). (**D**–**G**) Neutrophil markers (**D**) Lcn2 (*p* < 0.01), (**E**) Ly6g (*p* = 0.0737), (**F**) s100a8 (*p* < 0.05), and (**G**) s100a9 (*p* < 0.05) were elevated in burn compared to sham and (**I**–**L**) all positively correlated with plasma Ang-2 levels (*p* < 0.05). For two-group analysis, significance was analyzed by unpaired *t*-test and correlation assessed by simple-linear regression, * *p* < 0.05, ** *p* < 0.01, **** *p* < 0.0001.

Pulmonary neutrophil infiltration has been well characterized by our group and others within 6–24 h of burn injury [22,23,24,35]. In support of previous findings, we confirmed increased neutrophils within the lungs of burn mice in this study by histological analysis of H&E (*p* < 0.0001) (Figure 3A,B). Lungs of burn mice exhibited increased neutrophil chemokine C-X-C motif chemokine ligand 1 (Cxcl1) (*p* < 0.05) and increased neutrophil markers Lipocalin2 (Lcn2) (*p* < 0.01), Lymphocyte antigen 6 complex locus G (Ly6g) (*p* = 0.0737), s100a8 (*p* < 0.05), and s100a9 (*p* < 0.05) (Figure 3C–G). Each of these neutrophil markers significantly positively correlated with plasma Ang-2 levels (*p* < 0.05) (Figure 3H–L).

## 4. Discussion

For patients with large burns that survive initial resuscitation efforts, pneumonia remains the most common complication of their hospital course, with approximately half of burn patients developing pneumonia, ultimately contributing to 25% of burn-related mortality [3,4]. Previous studies have suggested that an imbalance between Ang-1 and Ang-2 plays a role in poor clinical outcomes in community-acquired pneumonia [10], acute respiratory distress syndrome (ARDS) [36,37], and mortality in critically ill and septic patients [36,37,38,39]. Elevations in Ang-2 and an imbalance in Ang-2/1 have been demonstrated to be predictive of mortality in burn patients [8]. Building on our similar recent findings linking early increases in Ang-2 and Ang-2/1 ratio to 30-day mortality in burn patients [9], this study aimed to investigate whether an elevation in Ang-2 and an imbalance in the Ang-2/1 ratio were observed at higher levels in patients who developed pneumonia. We then utilized a well-established murine model of scald burn injury to further evaluate Ang-2 and downstream mediators.

This study demonstrates that, on post-burn days 2 and 3, an early elevation of Ang-2 and an imbalance of Ang-2/1 are predictive of 30-day development of pneumonia in burn patients (Figure 1). Early evaluation of Ang-2 or the Ang-2/1 ratio may aid in prognostication for patients at risk of developing pneumonia during their hospital course but a larger cohort of patients with more patients developing pneumonia is crucial to understand if high Ang-2 is truly predictive of pneumonia development. In multivariable logistic regression adjusted for age, TBSA, and inhalation injury; higher Ang-2 on PBD2 and PBD3 were not significantly associated with increased odds of pneumonia, necessitating a larger study or multi-center study to determine if early elevations in Ang-2 are predictive of pneumonia development.

One factor likely contributing to pneumonia development in these patients is the incidence of inhalation injury (II). While only nine patients in this study sustained inhalation injury, the rate of II among those who went on to develop pneumonia was 40%. Further studies are warranted to determine if inhalation injury itself contributes to Ang-2 release, as the lungs are a highly vascularized organ lined with large quantities of endothelium [40]. In patients who developed pneumonia, Ang-1, Ang-2, and Ang-2/1 ratios were similar between those who did and did not sustain inhalation injury, but future studies are warranted to determine if direct insult to lung endothelium may contribute to elevations in Ang-2 levels.

The competitive interaction between Ang-1 and Ang-2 at the Tie2 receptor likely influences these outcomes, with Ang-2 promoting endothelial permeability and neutrophil infiltration. At homeostasis, Ang-1 interacts with TIE2 to inhibit the expression of Ang-2, ICAM-1, and VCAM-1 [13,15,16]. Under basal conditions, Ang-2 is sequestered within endothelial Weibel–Palade bodies from which Ang-2 is rapidly released upon damage and stress to endothelium [41]. Ang-2 competes with Ang-1 for binding to TIE2, resulting in a positive feedback loop, which upregulates expression of additional Ang-2 along with adhesion molecules ICAM-1 and VCAM-1 [17,34].

Our murine model of scald burn recapitulates an early elevation of circulating Ang-2 in burn patients (Figure 2A) [41]. We then utilized this model to evaluate Ang-2 and downstream mediators within the lungs. IHC demonstrated diffuse Ang-2 throughout the lungs of burn mice when compared to sham injury (Figure 2B). Pulmonary expression of the TIE2 receptor transcript Tek was significantly increased following burn, which positively correlated with plasma Ang-2 (Figure 2C,D). Ang-2 acts primarily on the TIE2 receptor, utilizing PI3K and ABIN2 for downstream signaling [34]. Tnip2 and Pik3r1, transcripts for ABIN2 and a subunit of PI3K, respectively, were significantly increased in the lungs following burn injury (Figure 2E,F), levels of which strongly positively correlated with plasma Ang-2 levels (Figure 2I,J). Ang-2 through its antagonist action on TIE2 has been established to increase levels of ICAM-1 and VCAM-1 in multiple disease states [34,42], which can be mitigated with Ang-1 agonism of TIE2 [16]. Expression of ICAM-1 and VCAM-1 were both significantly increased upon burn (Figure 2G,H), which also strongly correlated with plasma Ang-2 (Figure 2K,L). Together, this data provides evidence that increased Ang-2 may be acting through canonical TIE2 signaling mechanisms within the lungs following burn.

ICAM-1 and VCAM-1 on the endothelial lining both adhere to integrins on neutrophils, causing firm adhesion contributing to increased neutrophil extravasation into tissues [18,19,20,21]. Increased neutrophils in circulation and infiltration into tissues, including the lungs, has been well established following burn injury and thought to contribute to tissue damage and adverse outcomes [22,28,43,44]. We recapitulated established findings of increased neutrophils within the lungs following burn by evaluating H&E and expression of neutrophil markers (Figure 3A,B). We found increased neutrophils along with increased neutrophil chemokine Cxcl1 and neutrophil markers Lcn2, Ly6g, s100a8, and s100a9, which were all positively correlated with plasma Ang-2 (Figure 3C–L). Future studies are warranted to determine if inhibition of Ang-2 or a TIE2 agonist in the context of burn injury would decrease ICAM-1 and VCAM-1 and if this would result in a functional decrease in neutrophil infiltration into the lungs.

Development of pneumonia is a major contributor to morbidity and mortality following burn injury, which is difficult to prognosticate and there are no prophylactic treatments to address pneumonia development beyond strategies to mitigate risk of infection [5,6]. In this study, we demonstrate that an early elevation (day 2 and 3 after injury) of Ang-2 and the Ang-2/1 ratio both strongly correlate with the 30-day development of pneumonia. This provides support for further validation of Ang-2 and Ang-2/1 ratio as biomarkers for prognostication of pneumonia risk for burn patients.

Our murine studies show robust elevation of Ang-2, Tek, and downstream mediators within the lungs after burn injury (Figure 2 and Figure 3). These findings are observational and correlative. Future studies are necessary to determine if the pharmacological intervention blocking Ang-2 or enhancing Ang-1 activity on TIE2 are able to rescue vascular permeability and neutrophil infiltration into lungs and ultimately reduce mortality post-burn injury. Due to its role in angiogenesis and vascular permeability in the context of cancer metastasis, there are multiple studies utilizing antibodies that neutralize Ang-2 in preclinical oncologic models [45]. Studies of a specific TIE2 agonist AV-001 have shown efficacy in protection against both bacterial and viral pneumonia and may be useful in burn patients [46,47]. MT-001, a Tie2-agonistic antibody, has shown promise in vascular activation but has also shown off-target effects through sushi repeat-containing protein X-linked 2 (Srpx2) [48]. Another novel option for agonism of Tie2 is a lipid nanoparticle with mRNA cargo, such as mRNA-76 encoding human Ang-1, which has been shown to increase Ang-1-mediated Tie2 activation and decrease pulmonary vascular leakage in a murine model of lipopolysaccharide (LPS) challenge [49]. Given these promising applications, further studies are warranted to determine if modulation of the Ang–TIE2 axis would be beneficial in burn patients.

Together, this study provides support for further investigation of Ang-2, Ang-2/1, and TIE2 as targets for both prognostication and pharmaceutical intervention for pneumonia in burn patients.

## 5. Conclusions

In burn patients, an early elevation in Angiopoietin-2 and an imbalance of the Angiopoietin-2/1 ratio correlate with the development of pneumonia. In a murine model, burn injury increases Angiopoetin-2 and downstream signaling in the lungs, which may contribute to post-burn pulmonary dysfunction. Further studies are necessary to understand if Ang-2 levels can prognosticate pneumonia risk and if modulating the Angiopoietin–TIE2 axis can protect against pneumonia post-burn.

## Figures and Tables

**Table 2:** Multivariable logistic regression for pneumonia development.

Biomarker	OR #	SE #	CI #	*p* Value *
PBD2 Ang-1	1.095	0.519	0.399–3.24	0.861
PBD3 Ang-1	1.305	0.571	0.428–4.568	0.641
PBD2 Ang-2	5.229	0.874	1.109–39.096	0.0585
PBD3 Ang-2	100.639	2.442	3.278–102,009.561	0.0590
PBD2 Ang-2/1 Ratio	2.255	0.503	0.883–6.805	0.106
PBD3 Ang-2/1 Ratio	4.696	0.881	1.389–48.967	0.0792

# OR = odds ratio, SE = standard error, CI = 95% confidence interval. * Multivariable logistic regression adjusted for age, total body surface area (TBSA), and inhalation injury (II).

## Data Availability

The data presented in this study are available on request from the corresponding author due to the risk of re-identification inherent to small cohort clinical datasets.

## Abbreviations

The following abbreviations are used in this manuscript:

ABIN2	A20-binding inhibitor of NF-kb 2
AIS	abbreviated injury score
Ang-1	Angiopoietin-1
Ang-2	Angiopoietin-2
ARDS	acute respiratory distress syndrome
BCQP	Burn Care Quality Platform
BMI	body mass index
Cxcl1	C-X-C motif chemokine ligand 1
ELISA	Enzyme-Linked Immunosorbent Assay
H&E	hematoxylin and eosin
ICAM-1	intracellular adhesion molecule-1
ICU	intensive care unit
II	inhalation injury
Lcn2	Lipocalin2
LOS	length of stay
LPS	Lipopolysaccharide
Ly6g	Lymphocyte antigen 6 complex locus G
NET	Neutrophil Extracellular Trap
OR	Odds Ratio
PBD2	post-burn day 2
PBD3	post-burn day 3
PI3K	phosphoinositide 3-kinase
Pik3r1	phosphoinositide-3-kinase regulatory subunit 1
RT-qPCR	reverse transcription-quantitative polymerase chain
Srpx2	sushi repeat-containing protein X-linked 2
TIE2	Tyrosine kinase with immunoglobulin and epidermal growth factor homology domains 2
Tnip2	TNFAIP3 Interacting Protein 2
VAP	ventilator-associated pneumonia
VCAM-1	vascular cell adhesion molecule-1

## Appendix A

**Table A1:** qPCR TaqMan primers.

Target	Assay ID
Tek (TIE2)	Mm00443243_m1
Pik3r1	Mm01282781_m1
Tnip2	Mm00460482_m1
Icam1	Mm00516023_m1
Vcam1	Mm01320970_m1
Cxcl1	Mm04207460_m1
Lcn2	Mm01324470_m1
Ly6g	Mm04934123_m1
s100a8	Mm00496696_g1
s100a9	Mm00656925_m1
